# Supplementary material for: Supernumerary Head of the Biceps Brachii Muscle Influences the Topography of the Coracobrachialis and Biceps Brachii Muscles
Source: Medicina (Kaunas). 2024 Oct 22;60(11):1726. doi: 10.3390/medicina60111726 (PMC11596292; doi:10.3390/medicina60111726)

Supplementary table S1. Anatomical Location of the Muscle and Neurovascular Structures

	Mean (mm)	Std. deviation	Minimum	Maximum
Upper Limb	486.83	31.31	420	550
CBM proximal insertion	109.03	16.78	73	149
CBM distal insertion	147.36	16.04	114	203
BBM head	281.33	23.97	231	396
DBA	76.50	24.45	0	124
MNR	29.06	24.82	0	104
Point of CBM pierced by MCN	48.43	20.12	0	93

All locations were measured from the coracoid process, except for the short head of the biceps brachii muscle. BBM: biceps brachii, CBM: coracobrachialis muscle, DBA: deep brachial artery, MNR: median nerve root

Supplementary table S2. Gender difference of anatomical Location of the Muscle and Neurovascular Structures

	Male (n = 55)	Female	P value
CBM proximal insertion	115.54 ± 15.99	103.35 ± 15.45	< 0.001
CBM distal insertion	153.20 ± 15.60	142.25 ± 14.36	< 0.001
BBM head	294.50 ± 28.06	286.44 ± 20.97	0.099
DBA	77.96 ± 23.30	74.81 ± 25.85	0.517
MNR	29.67 ± 25.51	28.35 ± 24.25	0.789
Point of CBM pierced by MCN	48.75 ± 19.35	48.06 ± 21.15	0.865

All locations were measured from the coracoid process, except for the short head of the biceps brachii muscle. BBM: biceps brachii, CBM: coracobrachialis muscle, DBA: deep brachial artery, MNR: median nerve root

Supplementary figure S1. Representative image of supernumerary head of the biceps brachii muscle (BBM). Between two heads, superior head(*) was found. Infero-lateral head of BBM was shown in middle level of upper arm, and this cadaver had four heads of BBM. CBM, coracobrachialis muscle; MNR, median nerve root.

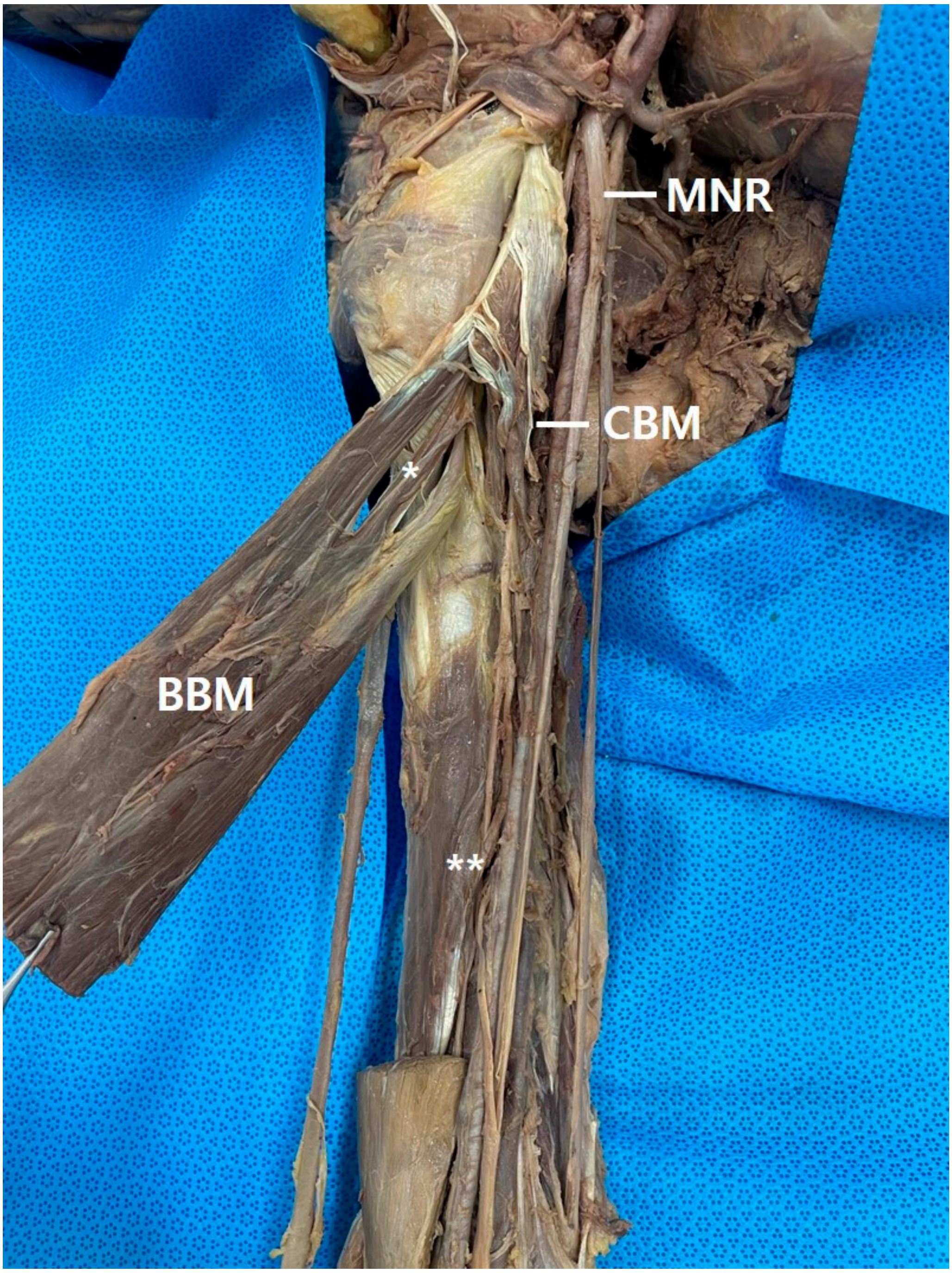

Supplement: Supplementary file 1 [file medicina-60-01726-s001.zip › medicina-3169787-supplementary.pdf]
